# A Study of Parallelism of the Occlusal Plane and Ala-Tragus Line

**DOI:** 10.5681/joddd.2009.027

**Published:** 2009-12-15

**Authors:** Katayoun Sadr, Makan Sadr

**Affiliations:** ^1^ Assistant Professor, Department of Prosthodontics, Faculty of Dentistry, Tabriz University of Medical science, Tabriz, Iran; ^2^ PhD Student of Anatomy, Faculty of Medicine, Tehran University of Medical Science, Tehran, Iran

**Keywords:** Occlusal plane, ala-tragus line, complete denture

## Abstract

**Background and aims:**

Orientation of the occlusal plane is one of the most important clinical procedures in prostho-dontic rehabilitation of edentulous patients. The aim of this study was to define the best posterior reference point of ala-tragus line for orientation of occlusal plane for complete denture fabrication.

**Materials and methods:**

Fifty-three dental students (27 females and 26 males) with complete natural dentition and Angel’s Class I occlusal relationship were selected. The subjects were photographed in natural head position while clenching on a Fox plane. After tracing the photographs, the angles between the following lines were measured: the occlusal plane (Fox plane) and the superior border of ala-tragus, the occlusal plane (Fox plane) and the middle of ala-tragus as well as the occlusal plane (Fox plane) and the inferior border of ala-tragus. Descriptive statistics, one sample t-test and independent t-test were used. P value less than 0.05 was considered significant.

**Results:**

There was no parallelism between the occlusal plane and ala-tragus line with three different posterior ends and one sample t-test showed that the angles between them were significantly different from zero (p<0.05). However, the supe-rior border of ala-tragus line had the lowest mean angle, 1.80° (3.12) and was almost parallel to the occlusal plane.

**Conclusion:**

The superior border of the tragus is suggested as the posterior reference for ala-tragus line.

## Introduction

Orientation of the occlusal plane is one of the most important clinical procedures in prosthodontic rehabilitation of edentulous patients and because of its effect on aesthetics, function and denture stability, it should be reconstructed as identical as possible to the occlusal plane of missing natural teeth.^1^

There are various methods that utilize intraoral and extraoral landmarks for orientation of the occlusal plane. The use of the ala-tragus line to orient the occlusal plane is advocated by some authors. However, there is some controversy on the exact points of references of the ala-tragus line.^2^

The Glossary of Prosthodontics Terms ^3^ states that the ala-tragus line runs from the inferior border of the ala of the nose to some defined point on the tragus of the ear, usually considered to the tip of the tragus. It does not stipulate which part of the tragus should be used as the posterior landmark.

Zarb and Bolender^4^ advocate that the occlusal plane should be parallel to the ala-tragus line posteriorly without defining or illustrating it. However, texts by Winkler,^5^ Rahn and Heartwell,^6^ and Boucher ^7^describe it as a line running at the inferior border of the ala of the nose to the superior border of tragus of the ear.

The aim of the present study was to define the best posterior reference point of the ala-tragus line for orientation of occlusal plane for complete denture fabrication.

## Materials and methods

In this cross-sectional study, fifty-three dental students (27 females and 26 males) with complete natural dentition and Angel’s Class I occlusal relationship were selected. Exclusion criteria were as follows:

1. Previous orthodontic and prosthodontic treatment and history of aesthetic surgery
2. Facial asymmetry and craniofacial anomaly
3. Overjet and overbite over 2 mm

Following case selection, the subjects were asked to hold a Fox plane, covered with dental wax, between their teeth (Fig 1a). Left profile photographs were taken with a digital camera (Sony F.707, 5- megapixel) with the subjects standing in natural head position. An adjustable tripod (Fujitsu Video tripod 2002) was used for adjusting the camera with the height of the Fox plane in the subjects. Then the photographs were traced (Fig 1b). Two investigators independently measured the angles between the following lines:

Figure 1. Subject in natural head position with a Fox plane (a). Tracing of the photograph, 1= FP-ATs, 2=FP-ATm, 3=FP-ATi, 4= FP (b).

**a:**
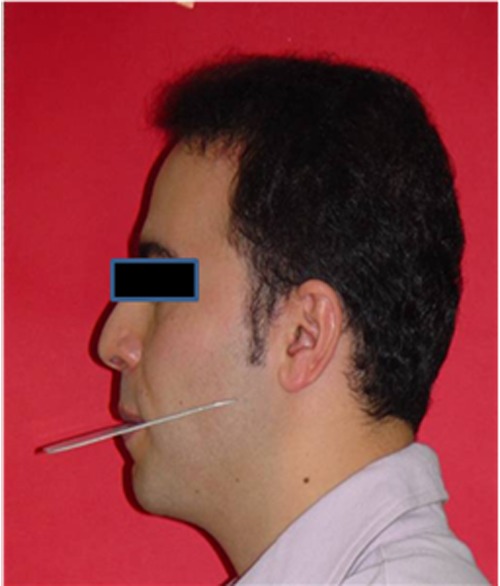

**b:**
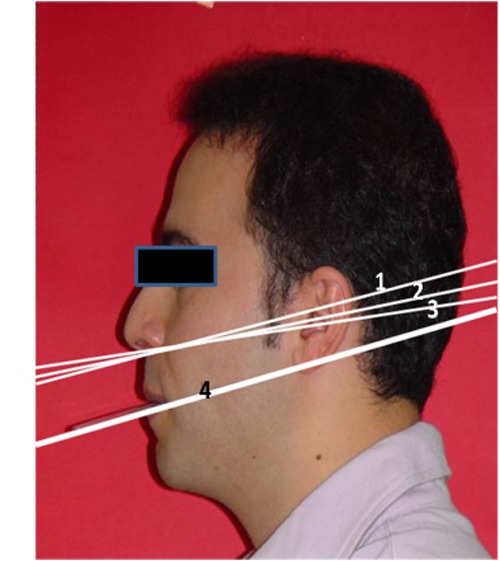

1. Occlusal plane (Fox plane) and the superior border of ala-tragus (FP-ATs)
2. Occlusal plane (Fox plane) and the middle of ala-tragus (FP-ATm)
3. Occlusal plane (Fox plane) and the inferior border of ala-tragus (FP-ATi)
4. Statistical analysis was carried out using SPSS software, Version 13. Descriptive statistics, one sample t-test and independent t-test were used. Test value was zero in one sample t-test, because it was supposed that the occlusal plane and the ala-tragus line should be parallel.

## Results

Mean values and standard deviations of the angles between the Fox plane and the ala-tragus with three posterior point locations are presented in Table 1. The angles between the occlusal plane and ATs, ATm, ATi, are significantly different from zero. It means no parallelism exists between the occlusal plane and the ala-tragus line. There is also no parallelism between the Fox plane and the ala-tragus with three posterior point locations in males and females; however, the results showed that in both sexes the superior border of the ala-tragus line had the lowest mean value and stronger tendency to be parallel to the Fox plane.

**Table 1:** Angles (means and SDs) and significance difference between Fox plane and ala-tragus line with three posterior point locations in male and female subjects

		All (n=53)				Female (n=27)				Male (n=26)		
Angle	Mean	SD	t	P	Mean	SD	t	P	Mean	SD	t	P
FP-ATs	1.801°	3.123	4.200	<0.001	2.870°	3.745	3.982	<0.001	0.692°	1.783	1.979	<0.059
FP-ATm	4.160°	3.893	7.779	<0.001	6.203°	3.698	8.716	<0.001	2.038°	2.849	3.648	<0.001
FP-ATi	5.839°	4.770	8.912	<0.001	8.370°	4.375	9.940	<0.001	3.211°	3.650	4.486	<0.001

FP-ATs: Fox plane - the superior border of ala-tragus; FP-ATm: Fox plane - the middle of ala-tragus; FP-ATi: Fox plane - the inferior border of ala-tragus.

## Discussion

Using ala-tragus line for establishing occlusal plane is a common method in the fabrication of complete dentures. However, there is some controversy on the posterior end of the ala-tragus line. Given these factors, it was decided to determine which one is paral

lel to the occlusal plane and could be use for orientation the occlusal plane: the superior border of ala-tragus, the central point of ala-tragus or the inferior border of ala-tragus.

According to the result of the present study, there is no parallelism between the occlusal plane and the ala-tragus line with three different posterior ends. The average angle between the occlusal plane and the ala-superior border of tragus was 1.80° (3.12); the average angle between the occlusal plane and the middle of ala-tragus was 4.16° (3.89); and the average angle between the occlusal plane and the inferior border of ala-tragus was 5.83° (4.77). The superior border of the ala-tragus line had the lowest mean angle (1.80°) (3.12) and is almost parallel to the occlusal plane.

Van Niekerk et al ^8^reported from a cephalometric study that the angle between the occlusal plane and the ala-tragus line was 2.45° (3.24) in denture-wearing subjects. They used the inferior border of tragus as the posterior end of the ala-tragus line because it could provide sufficient space for the arrangement of maxillary posterior teeth. However, similar to the results of the present study the inferior border of the ala-tragus line had the largest angle. The subjects of Van Niekerk’s study were edentulous and the different position of the posterior teeth in complete denture compared with the natural dentition could explain the differences in results.

Karkazis and Polysois^9^showed in a cephalometric study that natural and artificial occlusal planes are not parallel to the ala-tragus line. The average angles for natural and artificial teeth were 2.84° (3.45) and 3.25° (4.69), respectively. They used the center of the tragus as the posterior point for the ala-tragus line. Petricevic et al^1^ reported a 3.94° (5.57) angle between the occlusal plane and the Camper plane, which is consistent with the results of the present study. The difference between the results of the present study and the other studies could be explained by the use of different methods of study and points of measurement.

## Conclusion

According to the results of the present study, the superior border of the tragus is suggested as the posterior reference for the ala-tragus line.
